# The Importance of Posterior Hyaloid Removal in a Case of Vitrectomy for Floaters in High Myopia

**DOI:** 10.7759/cureus.60830

**Published:** 2024-05-22

**Authors:** Mohd Khairrudin M Mohd Sobri, Mae-Lynn Catherine Bastion, Chenshen Lam, Zairah Zainal Abidin

**Affiliations:** 1 Ophthalmology, Hospital Ampang, Kuala Lumpur, MYS; 2 Ophthalmology, Hospital Universiti Kebangsaan Malaysia, Kuala Lumpur, MYS; 3 Ophthalmology, Universiti Kebangsaan Malaysia Medical Centre, Kuala Lumpur, MYS

**Keywords:** vitrectomy, high myopia, floaters, myopic foveoschisis, ilm peel

## Abstract

A 61-year-old Malaysian Chinese man who has high myopia complained of both eye floaters. Spectral-domain optical coherence tomography (SD-OCT) of the macula showed bilateral posterior staphyloma with right eye (RE) foveoschisis without macula detachment, which had been stable for a seven-year follow-up. When bilateral YAG laser vitreolysis could not alleviate his symptoms, he underwent pars plana vitrectomy with the inducement of posterior vitreous detachment, first in the left eye, followed by the RE one month later. The best-corrected visual acuity for both eyes was 6/6, N5 two months postoperatively, and he was asymptomatic for floaters. However, six months postoperatively, he complained of metamorphopsia and worsening RE vision. Repeat OCT showed worsening of the foveoschisis bilaterally with left foveal detachment. The patient had to undergo a repeat vitrectomy with peeling of the internal limiting membrane (ILM) in bilateral eyes, which successfully restored his foveal architecture and alleviated his symptoms. This article highlights theimportance of preoperative OCT assessment of the fovea in patients undergoing vitrectomy for floaters, as staining and complete removal of posterior hyaloid with ILM peeling during vitrectomy may mitigate the progression of foveoschisis after core vitrectomy for floaters in myopic patients.

## Introduction

Myopic foveoschisis (MF) is characterized by an intraretinal cleavage or splitting of neurosensory retinal components in a myopic posterior staphyloma. It is one of the complications of high myopia. MF is a form of acquired retinoschisis [1]. MF may progress to foveal detachment, lamellar macular hole, full-thickness macular hole, and macular detachment. It may also be associated with the epiretinal membrane or vitreomacular traction (VMT). The separation of the retinal layers in MF is postulated to be due to inward traction caused by a progressive ectasia of the sclera in high myopia and the relative resistance to a stretch of the inner retinal layers and the retinal vessels, as retinal vessels are stiffer relative to the retinal layer. MF may also be due to incomplete or anomalous posterior vitreous detachment (PVD) combined with contraction of the residual attachment of the cortical vitreous over the retina. The tractional forces exerted by the layer of cortical vitreous may be stronger, given the preexisting globe ectasia and staphyloma and relatively stiff retinal vessels [2-4].

MF progresses slowly and is usually asymptomatic. It can remain stable for years without causing any visual disturbance. However, in some cases, it results in mild central blurring of vision when myopic foveal detachment occurs. If a lamellar hole occurs, there may be metamorphopsia and a further decrease in vision until moderate to severe central vision loss occurs with the development of a full-thickness macula hole. The central scotoma enlarges when macula detachment occurs. Clinical examination is hampered by the chorioretinal atrophy that often accompanies myopic maculopathy in the presence of posterior staphyloma in high myopia. Hence, spectral-domain optical coherence tomography (SD-OCT) is invaluable in assessing MF and is the only tool for staging.

Vitrectomy with internal limiting membrane (ILM) peeling is widely accepted and successful as a surgical treatment for MF. It is most successful at maintaining and restoring vision when performed at the foveal detachment stage and less successful when the macula hole has developed [5,6].

Vitreolysis for floaters has been found to be beneficial for the treatment of floaters, particularly in cases with Weiss ring types. However, in cases with diffuse or cloud floaters, treatment involves multiple sessions and often fails to relieve all the floaters. Hence, a vitrectomy may be performed for a more complete removal. A number of studies have highlighted the effectiveness of vitrectomy for floaters and patient satisfaction after pars plana vitrectomy (PPV) was done [7-10]. Typically, evidence indicates that a core PPV is often sufficient for these eyes [7]. Sebag et al. conclude that vitrectomy improves patients’ floaters and improves contrast sensitivity, which is reduced by floaters, thus improving patients’ quality of life. They performed PPV without inducing PVD to reduce the risk of retinal tears from vitrectomy. The results were satisfactory, with no cases developing retinal breaks and only one case developing significantly disturbing floaters, which required repeated PPV [7].

A number of retrospective case reports have described repeated surgery for progression in MF despite vitrectomy [11,12]. Most of the cases that required repeated PPV were done without ILM peeling; in all these cases, none of them underwent PPV for floaters, unlike our case.

This case report aims to describe the progression of MF following bilateral sequential vitrectomy for floaters in high myopia, which has not been previously described.

## Case presentation

**Figure 1:**
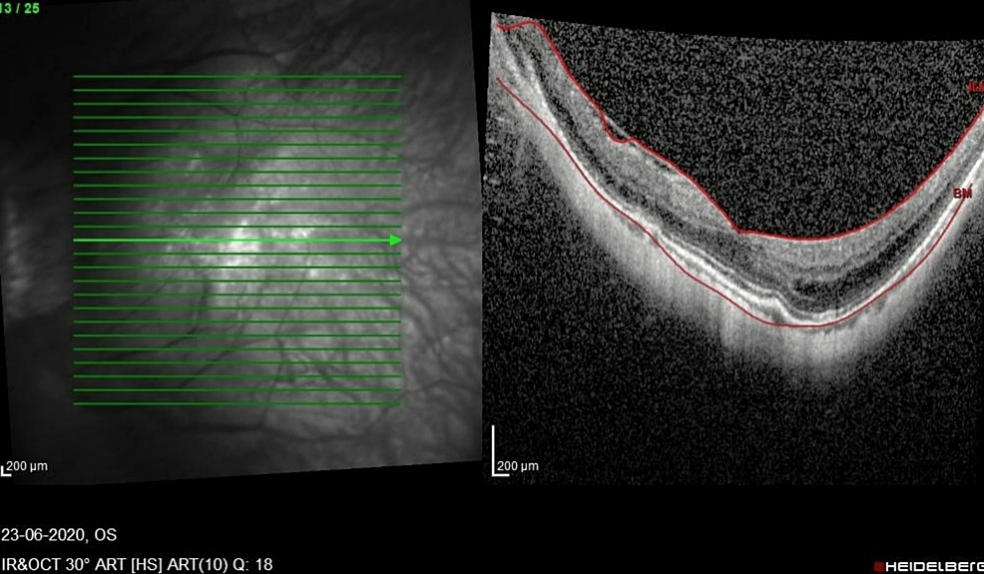
SD-OCT macula of the LE prior to the first vitrectomy. LE, left eye; SD-OCT, spectral-domain optical coherence tomography

**Figure 2:**
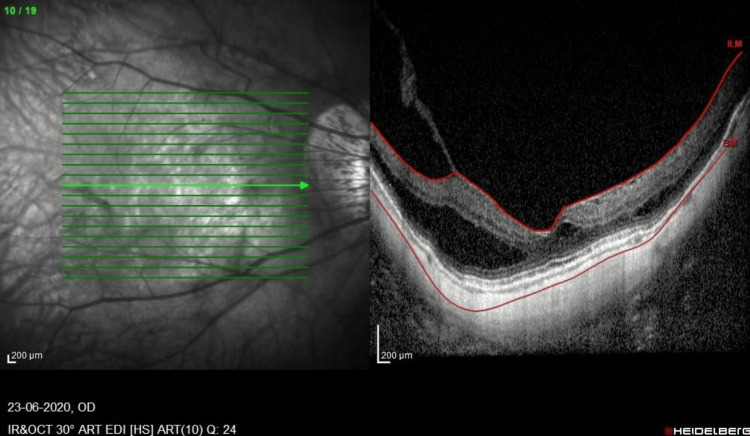
SD-OCT macula of the RE prior to the first vitrectomy showing foveoschisis with VMT. There is a minimal epiretinal membrane. RE, right eye; SD-OCT, spectral-domain optical coherence tomography; VMT, vitreomacular traction

A 61-year-old high-myopic Malaysian Chinese man with no known medical illness presented seven years ago with mild metamorphopsia in his right eye (RE) for a one-year duration. He had a history of bilateral uneventful phacoemulsification with intraocular lens implantation two years prior. On examination, his RE vision was 6/12, N6, and his left eye (LE) vision was 6/18 with pinhole 6/12, N8 with right foveoschisis and Fuch spot, and bilateral posterior staphyloma. He was followed up with no progression over the course of seven years. He then complained of bilateral floaters for several months. The anterior segment examination was unremarkable, with stable intraocular lenses. Posterior segment examination showed both eyes had PVD with vitreous syneresis, and no retinal tear or break was seen. SD-OCT (SPECTRALIS®, Heidelberg Engineering GmbH, Heidelberg, Germany) revealed bilateral stable MF, and the LE had a center-sparing epiretinal membrane (Figure 1, Figure 2).

The patient then underwent a LE 27G PPV under local anesthesia (LA) for floaters, during which a core vitrectomy was performed following manual inducement of PVD. After one month, a RE 27G PPV/endolaser under LA was performed with the manual inducement of PVD using the vitrectomy cutter on suction. Intraoperative findings included one small retinal hole seen at the inferior temporal retina at the edge of the posterior staphyloma with no retinal detachment. A gentle laser was applied to this intraoperatively.

**Figure 3:**
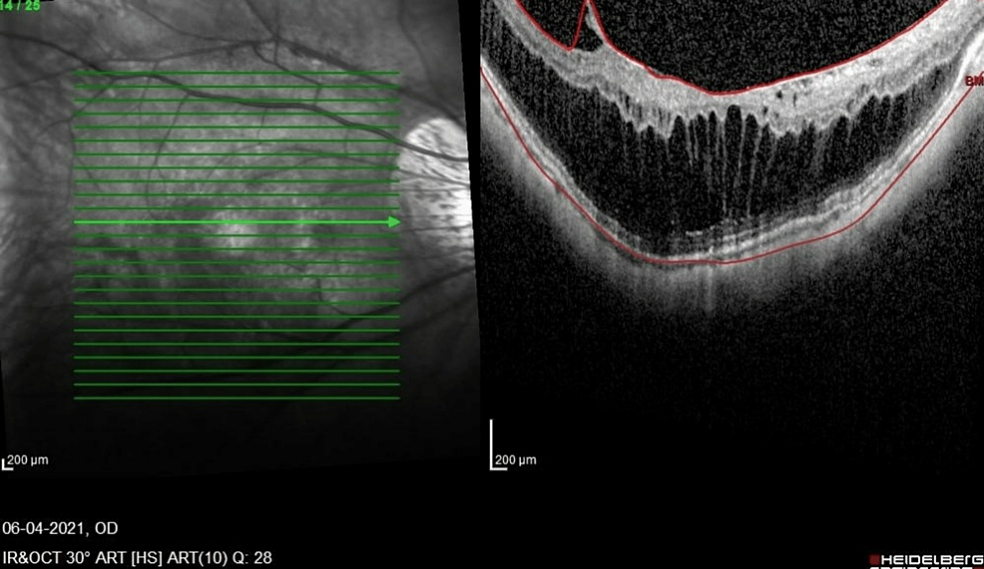
OCT macula of the RE six months post-vitrectomy. Progression of MF without foveal detachment and progression of the epiretinal membrane with traction is noted. MF, myopic foveoschisis; OCT, optical coherence tomography; RE, right eye

**Figure 4:**
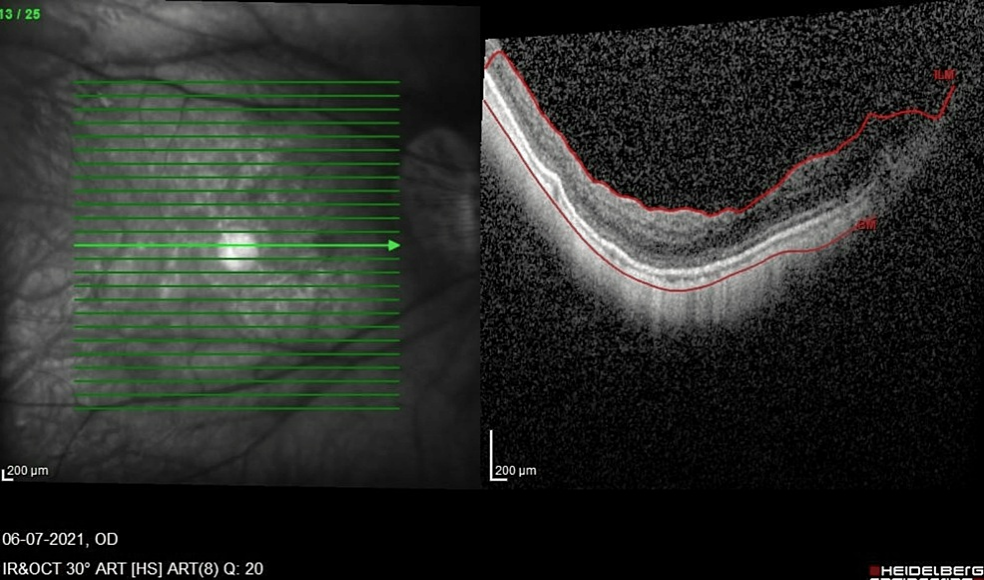
OCT macula of the RE six weeks post-vitrectomy revision shows resolution of traction and schisis. OCT, optical coherence tomography; RE, right eye

Two months post-RE vitrectomy, bilateral best-corrected visual acuity was 6/6, N6, with no complete relief from floaters. However, during the six-month postoperative follow-up, he complained of mild blurring of vision with a gradual worsening of metamorphopsia. His visual acuity at this point was 6/9.5, ph 6/9.5, and N10. The OCT macula of the RE showed progression of MF and VMT with the epiretinal membrane (Figure 3). He then underwent RE revision 25G vitrectomy with preservative-free triamcinolone staining of the posterior hyaloid remnant, dye-assisted (Twin membrane blue, Brand Alchimia, Italy) membrane, and ILM peeling to the edge of the posterior staphyloma. Intraoperatively, a taut posterior hyaloid was removed, and a small macula hole was suspected due to the presence of subretinal dye migration. Hence, 22% non-expansile sulfur hexafluoride (SF6) gas was injected, and the patient adopted a face-down posture for one week. After gas resorption, his RE OCT showed relief of the central traction and MF, and after six weeks, there was a better inner segment and outer segment (IS/OS) junction with a best-corrected visual acuity of 6/7.5, N8 (Figure 4) at three months postoperation. His metamorphopsia also improved.

**Figure 5:**
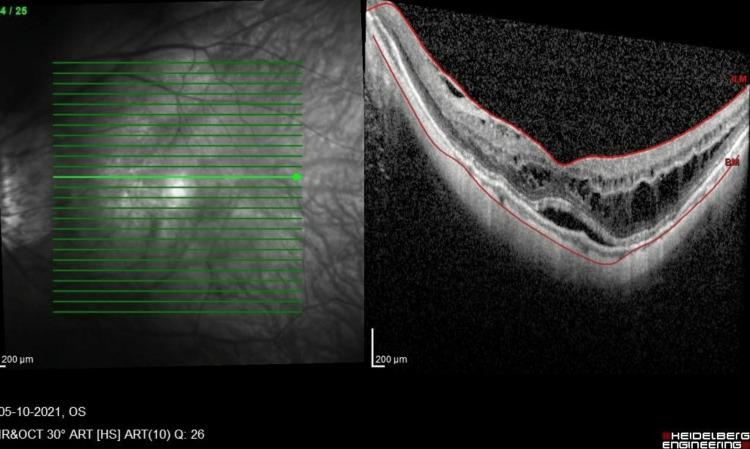
OCT macula of the LE 12 months after the first vitrectomy showing foveal detachment with mild foveoschisis. LE, left eye; OCT, optical coherence tomography

**Figure 6:**
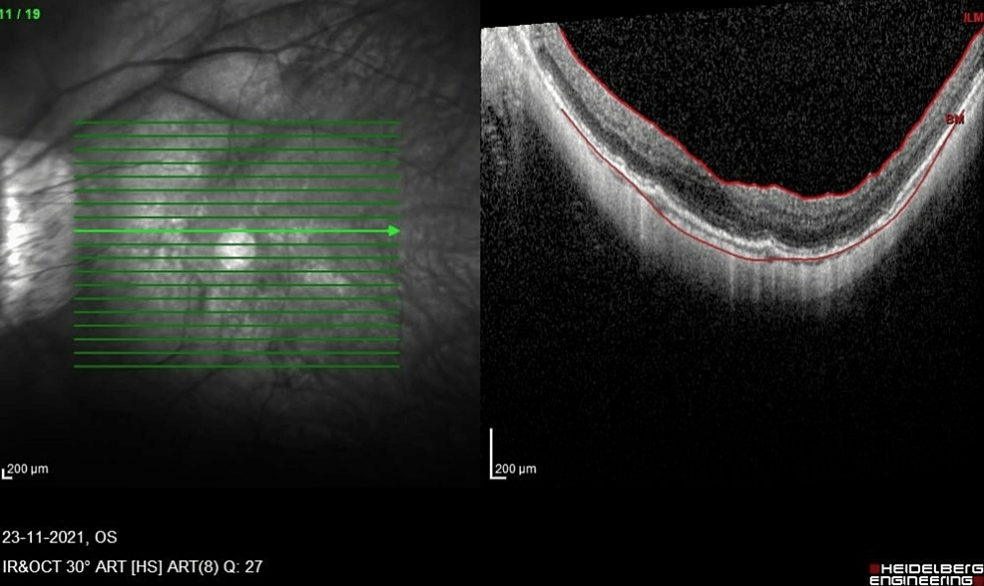
OCT macula of the LE six weeks post-revision vitrectomy. LE, left eye; OCT, optical coherence tomography

Interestingly, in the case of his LE, 12 months after the initial vitrectomy, a similar progression began to occur, with the LE developing a progression of foveoschisis leading to foveal detachment (Figure 5). He was asymptomatic, with the best-corrected vision of 6/7.5, N5. Nevertheless, he underwent LE revision 23G PPV with membrane and ILM peeling with Brilliant Peel® Dual Dye (Fluoron GmbH, Ulm, Germany). Peeling was performed using 23G ILM peeling forceps, as 25G forceps were not long enough. His OCT normalized postoperation (Figure 6), with the best-corrected visual acuity of 6/9.5, N5 at six weeks postoperation. The OCT remained stable up to the final follow-up three months after the surgery.

## Discussion

Floaters can cause disturbing visual symptoms and reduce contrast sensitivity in certain people, especially those who are young myopes, pseudophakic, and whose work needs fine and sharp visual acuity. This visual disturbance can disturb the quality of life, so they seek medical attention. As people age, the initial homogenous and transparent vitreous body undergoes structural changes that can cause reduced vitreous body homogeneity. This is due to liquefaction within the vitreous body; thus, collagen cross-linking can occur, which produces aggregation of the vitreous collagen framework [13,14]. These aggregates disturb light transmission through the vitreous and can cause what we call floaters, and if they are near the retina, they can cause an entoptic phenomenon, namely the light scattering vitreous floater. Most people can neuroadapt to their floaters, but a small minority of people still find them extremely disturbing to their vision and lifestyle [14].

Floaters that cause visual disturbance can be treated by Nd:YAG laser vitreolysis or PPV. In our case, the initial treatment for our patient was a YAG laser treatment in a single session. The floaters did not improve sufficiently after YAG laser treatment due to the nature of his floaters, which were multiple and cloudy. Delaney et al. found that YAG laser treatment brought moderate relief, with up to 54% of the patients experiencing no relief. On the other hand, 93.3% of patients who underwent vitrectomy experienced full symptomatic relief in their study [15]. However, this has to also be viewed from the perspective of the risks of the procedure. YAG laser vitreolysis is relatively safer, with low rates of complications. On the other hand, vitrectomy is invasive and carries a risk of retinal tears, retinal detachment, cataract formations, cystoid macular edema, endophthalmitis, and persistent floater symptoms [7,14,16]. Tan et al. reported 16.4% of cases of iatrogenic retinal breaks following vitrectomy for persistent floaters. They found that there is a correlation between PVD induction and retinal breaks [16].

MF is a fairly common disease in highly myopic patients and usually affects the middle-aged population [3]. OCT, particularly spectral-domain macula OCT, is required for a definitive diagnosis. MF is essentially a tractional disease that is generated from various components. The vitreous cortex causes inward traction to the retina, as shown in Figure 3 and Figure 4 in our patient. An epiretinal membrane may also generate traction. The rigidity of the ILM and the retinal vascular traction are also implicated in the pathogenesis of MF. The ILM is less flexible and can cause direct inward traction, and retinal vascular traction is postulated based on OCT findings, the so-called retinal microfolds [17]. The retinal vessels are less flexible and cannot be stretched as much as the other retinal components. Thus, MF is caused by multiple factors and can be regarded as a split between the flexible outer retina and the inflexible inner retina.

Hence, when there is a progression of MF to foveal detachment, vitrectomy is warranted. Vitrectomy in MF patients remains challenging due to the accompanying long axial length and difficulty in manipulating the instrument. As noted in the LE of this patient, the 25G ILM peeling forceps are not long enough to reach the posterior pole in highly myopic eyes such as this case. This is why routine peeling of the ILM is not performed routinely in all cases of vitrectomy for floaters, especially those with high myopia without MF. Yeh et al. reported three cases of highly myopic eyes that underwent vitrectomy without ILM peel and reported that it was effective for treating MF in their 12-month follow-up [18]. However, despite the core vitrectomy, the MF in our patient progressed. This is most likely due to the presence of vitreoschisis and a retained layer of posterior hyaloid, which is adherent to the retina. Staining the vitreous with materials such as triamcinolone would have helped visualize this layer. The good preoperative vision of the patient and the challenge of membrane peeling in such long axial length eyes were two of the factors in the initial surgery for floaters, and the surgeon did not proceed with ILM peeling during this surgery.

The vitreous structure in highly myopic eyes is unusual, as there may be vitreoschisis, which appears as a layer of cortical vitreous, adherent to the surface of the retina, separate from the core vitreous. This is likely to have been the cause of the retained and taut posterior hyaloid in this patient. This layer of vitreous is not always visualized on OCT, and despite the inducement of PVD with the vitrectomy cutter, it can be missed. The use of triamcinolone to highlight the cortical vitreous has been described in a few studies as it eases and helps to ensure the complete removal of the vitreous [12,19-21]. The short duration of its usage should not cause elevation of intraocular pressure or cataracts, as it is removed at the end of the surgery. This staining with triamcinolone should be done routinely even during vitrectomy for floaters in highly myopic eyes, with attention paid to removing the posterior hyaloid completely. We have found that a core vitrectomy is insufficient in such eyes.

Interestingly, the LE of this patient did not display significant MF (Figure 4), unlike the RE (Figure 3) on OCT. Nevertheless, foveal detachment still occurred. The onset is also much later than the RE. This highlights the role of the cortical vitreous remnant in inducing anterior-posterior traction in the absence of any OCT evidence of VMT or membranes. An increase in axial length after PPV is another possible factor, adding to the anterior-posterior length and stretch. Fortunately, ILM peeling with stain was successful in restoring the anatomy in both eyes with timely surgery, performed before the macular hole developed. Furthermore, a macula buckle was not required in this case. This decision to operate when fovea detachment has just occurred prior to a drop in vision is supported in various studies on the timing of vitrectomy in MF patients [20,22,23]. ILM peeling and gas tamponade during vitrectomy remain controversial, although there are reports of successful anatomical and visual outcomes without ILM peeling and/or gas tamponade [18,24-28].

Another interesting feature of the case is the long duration of six months to one year during which the progression in MF is seen. This highlights the need to follow up on high-myopia patients after vitrectomy, especially if triamcinolone was not used to ensure complete posterior hyaloid removal or if there is a residual epiretinal membrane. Counseling for myopic patients regarding the progression of MF and the potential of MF should also be provided prior to surgery for floaters.

## Conclusions

Our case shows the importance of complete removal of the posterior hyaloid during vitrectomy for floaters in a patient with a history of high myopia to prevent the progression of stable MF or the development of MF, which can progress to foveal detachment. This may reduce the need for a second procedure. The progression of MF in any myopic patient undergoing vitrectomy for floaters should also be discussed during preoperative counseling. Vitrectomy with dye-assisted membrane and ILM peeling is successful in restoring anatomy and function in a patient with the progression of MF to foveal detachment after vitrectomy for floaters. The choice of PPV gauge should take into account the axial length of the patient due to limitations with instrument length when ILM peeling is needed.
